# Lurbinectedin in Refractory Diffuse Malignant Peritoneal Mesothelioma: Report of Two Cases

**DOI:** 10.3389/fonc.2021.704295

**Published:** 2021-06-18

**Authors:** Louis Gros, Petr Szturz, Antonella Diciolla, Volker Kirchner, Solange Peters, Niklaus Schaefer, Martin Hubner, Antonia Digklia

**Affiliations:** ^1^ Department of Oncology, Lausanne University Hospital, University of Lausanne, Lausanne, Switzerland; ^2^ Department of Oncology, Clinique de Genolier, Genolier, Switzerland; ^3^ Department of Nuclear Medicine and Molecular Imaging, Lausanne University Hospital, University of Lausanne, Lausanne, Switzerland; ^4^ Department of Visceral Surgery, University Hospital of Lausanne, Lausanne, Switzerland

**Keywords:** peritoneal mesothelioma, lurbinectedin, case report, palliative chemotherapy, peritoneal tumor, durable response

## Abstract

Mesothelioma is a malignancy of serosal membranes. Parietal pleura is the most common site, with peritoneum being the second most frequent location. Malignant peritoneal mesothelioma (MPM) is a rare and aggressive disease. The prognosis is often very poor with median overall survival ranging from 6 to 18 months in patients who are not candidates for cytoreductive surgery (CRS) with hyperthermic intraperitoneal chemotherapy (HIPEC) due to non-resectable disease or comorbid conditions. For patients with resectable disease, CRS and HIPEC have become the standard of care. However, for patients with unresectable malignant mesothelioma there is unfortunately no effective systemic treatment beyond the first line. Based on the results of a recent phase II trial, lurbinectedin has clinical activity and acceptable toxicity in the second- and third-line treatment of malignant pleural mesothelioma. However, until present, no data have been available for patients with MPM and for patients who become refractory after multiple treatment lines. We report on two patients with metastatic MPM who achieved durable disease control of 10+ and 8 months with lurbinectedin in the fourth and fifth treatment line, respectively.

## Background

Malignant mesothelioma arises from mesothelial cells of the pleural, peritoneal, or pericardial linings. The pathogenesis of all forms of mesothelioma is strongly associated with toxic exposure to industrial pollutants, especially asbestos (1). Diffuse malignant peritoneal mesothelioma (DMPM) is an understudied disease because the majority of studies have been conducted predominantly in patients with the most common pleural variant. Given its rarity, treatment recommendations have been mostly based on single institutional retrospective reports or on indirect evidence (extrapolation) from trials performed in pleural mesothelioma (2). Patients with potentially resectable disease should be offered cytoreductive surgery (CRS) (3) and hyperthermic intraperitoneal chemotherapy (HIPEC) (4, 5). In patients who are not eligible for surgery or intraperitoneal chemotherapy, the most effective alternative is systemic chemotherapy combining pemetrexed with either cisplatin or carboplatin (6). As the efficacy of first-line chemotherapy remains poor, numerous studies have been carried out to improve the outcomes but without satisfactory results. A recent phase II trial of lurbinectedin in relapsed pleural mesothelioma showed a progression-free survival (PFS) rate of 52.4% at 12 months (7). Here, we report on good tolerability and efficacy of lurbinectedin in two patients with heavily pretreated DMPM.

## Case 1

**Figure 1:**
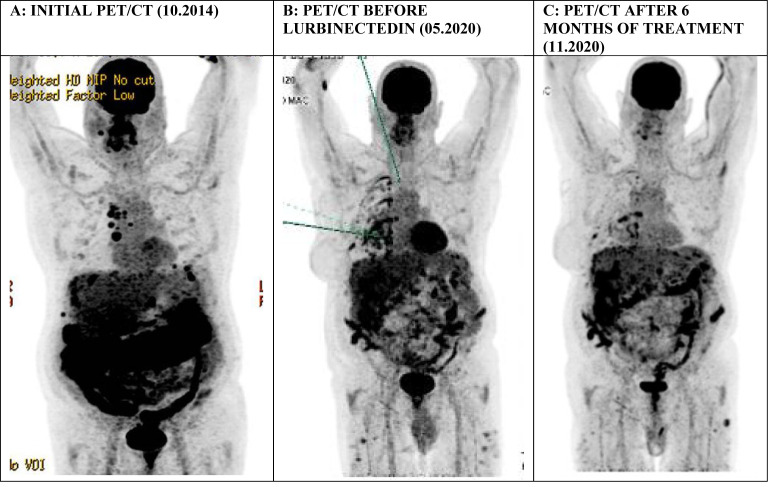
A series of PET/CT scans (Maximum Intensity Projections) in the first patient showing: **(A)** signs of peritoneal carcinomatosis in the subdiaphragmatic space infiltrating the large omentum and associated with ascites, in addition to supradiaphragmatic laterocervical and mediastinal lymphadenopathies, **(B)** persisting multiple peritoneal implant lesions at the time of treatment initiation, **(C)**  a significant improvement of the previously described peritoneal lesions with an overall regression of their metabolic activity.

In October 2014, a 66-year-old male, with a history of atrial fibrillation and 40 pack-years of smoking, presented to the emergency department complaining of chest pain. Positron emission tomography/computed tomography (PET/CT) revealed pleuropericardial effusion with multiple mediastinal, internal mammary, and right costo-diaphragmatic angle adenopathies as well as signs of peritoneal carcinomatosis infiltrating the large omentum and associated with ascites (**Figure 1A**). A biopsy of the left cardiophrenic nodule was performed. Histopathology demonstrated the presence of malignant epithelioid mesothelioma. The initial PET-CT showed an involvement of the peritoneal and pleural cavities. The disease being very limited extraperitoneally, the diagnosis of DMPM was retained after multidisciplinary discussion in our tumor board.

The patient was treated with three lines of systematic therapy including immune checkpoint inhibitors and complemented with intraperitoneal aerosol chemotherapy (**Table 1**).

**Table 1:** Clinical characteristics of both patients and overview of treatment lines.

	Case 1	Case 2
**Age**	66	37
**Sex**	male	male
**Histology**	epithelioid	Epithelioid
**Stage at initial diagnosis**	Stage IV	Stage I
PD-L1*	2%	N/A
**Microsatellite stability status**	MSS	N/A
		
**Neoadjuvant chemotherapy**	no	cisplatin and pemetrexed
**Surgery**	no	complete cytoreductive surgery and HIPEC
**Intraperitoneal aerosol chemotherapy**	no	4 administrations
**1st line treatment**	carboplatin and pemetrexed, maintenance pemetrexed	pemetrexed
**Total cycles**	6 cycles	3 cycles
**Best response**	stable disease	progressive disease
**Intraperitoneal aerosol chemotherapy**	13 administrations	no
**2nd line treatment**	nivolumab	pembrolizumab
**Total cycles**	5	xxx
**Best response**	progressive disease	progressive disease
**3rd line treatment**	vinorelbine and gemcitabine	ipilimumab and nivolumab
**Total cycles**	8	xxx
**Best response**	progressive disease	progressive disease
**4th line treatment**	lurbinectedin	vinorelbine and gemcitabine
**Best response**	stable disease	progressive disease
**5th line treatment**	–	lurbinectedin
**Best response**		stable disease
**Time to further progression**	–	8 months

PD-L1, programmed cell death ligand 1, N/A, not available.

^*^Tumour Proportion Score.

After the failure of vinorelbine and gemcitabine in the third line, we proposed a new palliative line based on lurbinectedin in analogy to pleural mesothelioma as part of a compassionate use program. The patient accepted our proposal and we began the treatment in May 2020. At that time, he only had intermittent abdominal pain of grade 1-2 according to Common Terminology Criteria for Adverse Events (CTCAE) version 4.0. His performance status was 0 as assessed with the Eastern Cooperative Oncology Group (ECOG) scale. A PET/CT examination at baseline showed progression of multiple peritoneal implant lesions and an overall metabolic progression of a diffuse pleural affection on the right side (**Figure 1B**).

The recommended starting dose of lurbinectedin is 3.2 mg/m^2^ every three weeks (q3wk). However, given that the patient had renal impairment (with estimated glomerular filtration rate of 33 ml/min), the dose was reduced to 50% (i.e., 1.6 mg/m^2^ q3wk).

The patient’s tolerance to the treatment was very good with grade 1 asthenia, grade 1 diarrhea, and grade 1 abdominal pain that could be easily managed with standard supportive measures. His blood count remained normal. Restaging PET/CT examinations performed at 3, 6, and 9 months of treatment initiation showed an overall metabolic regression of the previously described pleural and peritoneal lesions (**Figure 1C**). At the last clinical visit in March 2021, more than 6 years after the primary diagnosis, no signs of progression were noted, and the patient continued lurbinectedin with good treatment compliance.

## Case 2

In February 2016, a 37-year-old male, with no past medical history, presented with abdominal pain. PET/CT scan imaging showed a mass of the abdominal wall adjacent to the hepatic segment VI. A biopsy of the nodule was performed. Histopathology demonstrated the presence of malignant epithelioid mesothelioma. Exploratory laparoscopy confirmed the presence of DMPM which was deemed resectable. The patient underwent complete CRS and HIPEC. Unfortunately, several months later the disease relapsed and only palliative systemic therapy could be proposed (**Table 1**). In July 2019, the disease progressed after 2 months of the fourth-line gemcitabine/vinorelbine treatment. At that time, the patient was in very good clinical condition presenting with only grade 1 asthenia and grade 1 decreased appetite, which in the absence of an open clinical trial, allowed us to delay a further treatment line until May 2021, with the patient accepting our proposal to participate in the compassionate use program with lurbinectedin. The treatment was administered intravenously at the standard dose of 3.2 mg/m^2^ q3wk.

The patient showed very good tolerance to the treatment with only grade 1 asthenia and grade 1 abdominal pain, which were easily manageable with standard measures. His blood count remained normal.

PET/CT imaging performed after 3 months of treatment showed an overall metabolic regression of the previously described pleural and peritoneal lesions, which was maintained at 6 months. Unfortunately, at 8 months after the first lurbinectedin administration, the disease progressed.

To date, the patient is still alive with no substantial clinical deterioration.

## Discussion

Lurbinectedin is an analogue of trabectedin and a selective inhibitor of oncogenic transcription that binds preferentially to guanines located in the GC-rich regulatory areas of DNA gene promoters (8, 9). By preventing binding of transcription factors to their recognition sequences, the drug inhibits oncogenic transcription and leads to tumor cell apoptosis (10). It also affects the tumor microenvironment by inhibiting activated transcription in tumor-associated macrophages (11). Lurbinectedin has shown very promising clinical activity in a broad range of clinical trials (12). In June 2020, based on the results from a phase II trial (study B-005; NCT02454972), the US Food and Drug Administration (FDA) granted accelerated approval to lurbinectedin for adult patients with metastatic small-cell lung cancer with disease progression on or after platinum-based chemotherapy (12). The Swiss Group for Clinical Cancer Research (SAKK) 17/16 trial, a phase II single-arm study, was designed to evaluate lurbinectedin efficacy and safety in patients with progressive malignant pleural mesothelioma (7). The primary endpoint of PFS at 12 weeks was met by 52.4% of patients; median PFS and median overall survival were 4.1 months and 11.1 months, respectively. There was one complete and one partial remission, while stable disease was obtained in 20 patients, leading to an overall response rate of 4.8% and a disease control rate of 52.4%. The duration of disease control was 6.6 months, and the median number of completed cycles was 5.

To the best of our knowledge, this is the first report on efficacy of lurbinectedin in refractory metastatic MPM. Lurbinectedin treatment led to a rapid improvement of symptoms in both patients corresponding to a decrease of metabolic activity of the peritoneal lesions. The first patient is still under treatment 10 months after starting lurbinectedin. As for the second patient, he had a PFS of 8 months, thus higher than the median PFS observed in the SAKK 17/16 trial.

Furthermore, contrary to the SAKK 17/16 study enrolling patients who had progressed on or after one line of chemotherapy and one line of immunotherapy, our two patients were heavily pretreated with up to 4 lines of systemic treatment, including immune checkpoint inhibitors.

However, we note long survival in both cases, compared to what is expected in the context of advanced DMPM. This longevity can be partly explained by the good general condition of our two patients, a potentially non-aggressive histology, the performance of multiple lines of treatment and the experience of the surgical team and the hospital. These reasons may also partly explain the good response to lurbinectedin treatment.

In conclusion, the durable clinical response and good tolerance to lurbinectedin in these two cases suggest the utility of this drug in DMPM but need to be confirmed in a well-designed prospective clinical trial.

## Learning Points

- To date, there has been no established treatment strategy for patients with metastatic malignant peritoneal mesothelioma, and their prognosis remains very poor.
- Lurbinectedin is an analogue of trabectedin, demonstrating very promising clinical activity in a broad range of clinical trials.
- We report on two patients with repeatedly relapsing malignant peritoneal mesothelioma who achieved durable disease control with lurbinectedin in the fourth and fifth line, respectively.

## Data Availability Statement

The original contributions presented in the study are included in the article/supplementary material. Further inquiries can be directed to the corresponding authors.

## Ethics Statement

Written informed consent was obtained from the individual(s) for the publication of any potentially identifiable images or data included in this article.

## Author Contributions

Writing – LG, ADig. Review & editing: all authors. All authors contributed to the article and approved the submitted version.

## Conflict of Interest

The authors declare that the research was conducted in the absence of any commercial or financial relationships that could be construed as a potential conflict of interest.
